# Rectifying long-standing misconceptions about the ρ statistic for molecular dating

**DOI:** 10.1371/journal.pone.0212311

**Published:** 2019-02-19

**Authors:** Vincent Macaulay, Pedro Soares, Martin B. Richards

**Affiliations:** 1 School of Mathematics and Statistics, University of Glasgow, Glasgow, United Kingdom; 2 CBMA (Centre of Molecular and Environmental Biology), Department of Biology, University of Minho, Campus de Gualtar, Braga, Portugal; 3 Institute of Science and Innovation for Bio-Sustainability (IB-S), University of Minho, Campus de Gualtar, Braga, Portugal; 4 Instituto de Patologia e Imunologia Molecular da Universidade do Porto (IPATIMUP), Porto, Portugal; 5 Department of Biological Sciences, School of Applied Sciences, University of Huddersfield, Queensgate, Huddersfield, United Kingdom; SOKENDAI (The Graduate University for Advanced Studies), JAPAN

## Abstract

When divided by a given mutation rate, the ρ (rho) statistic provides a simple estimator of the age of a clade within a phylogenetic tree by averaging the number of mutations from each sample in the clade to its root. However, a long-standing critique of the use of ρ in genetic dating has been quite often cited. Here we show that the critique is unfounded. We demonstrate by a formal mathematical argument and illustrate with a simulation study that ρ estimates are unbiased and also that ρ and maximum likelihood estimates do not differ in any systematic fashion. We also demonstrate that the claim that the associated confidence intervals commonly estimate the uncertainty inappropriately is flawed since it relies on a means of calculating standard errors that is not used by any other researchers, whereas an established expression for the standard error is largely unproblematic. We conclude that ρ dating, alongside approaches such as maximum likelihood (ML) and Bayesian inference, remains a useful tool for genetic dating.

## Introduction

Archaeogenetics has been described as “the study of the human past using the techniques of molecular genetics” [1]. Mitochondrial DNA (mtDNA), particularly when analysed phylogeographically, was pivotal to the pioneer phase of archaeogenetics [2] and continues to play an important role, even as ancient DNA and genome-wide studies, which now allow for direct checking of age estimates and dispersal models, have become central [3,4]. The value of mtDNA is due to its high mutation rate, allowing the accumulation of diversity within the time frame of recent human evolution, and lack of recombination, allowing the reconstruction of extremely well-resolved phylogenetic trees. More particularly, alongside the paternally inherited Y-chromosome variation, the maternally inherited mtDNA is invaluable for assessing sex-specific dispersal patterns, which are now understood to have had a major impact in recent prehistory [5].

Of course, like the male-specific part of the Y chromosome (MSY), the mtDNA is inherited as single locus, subject to the vagaries of one realization of genetic drift. They capture merely some shadow of human evolution. For example, neither preserves signals of admixture with archaic species of human, which required autosomal data to detect it [6]. In the future, haplotype blocks within the autosomes should provide a goldmine of phylogeographic insight. Nevertheless, due to the high density of non-recombining markers they carry, the phylogeography of these two genetic systems are uniquely fine-grained, with genealogical trees that reflect processes in human history in important ways. For example, the discovery, based on mtDNA variation, of an origin for modern humans in Africa by around 200 ka [7,8] followed by an out-of-Africa migration between 60 and 70 ka, has been refined and substantiated by other lines of evidence over the last three decades [9–11].

This molecular dating, whether based on the mtDNA, the MSY or haplotype blocks elsewhere in the genome, is based on a calibrated molecular clock. Properties of the mtDNA clock have been painstakingly worked out over many years [12], including the evaluation of and correction for the effects of purifying selection on the time estimates [13–16]. Some consensus seems to have been achieved, as current molecular clock estimates calibrated using very different approaches, using paleontological data [13,14], archaeological data [4] and radiometrically dated ancient DNA sequences [1,3,8,17–19] now provide largely compatible results. Moreover, the most widely used whole-mtDNA clock at present [14] has provided reliable, independently verifiable age estimates for the settlement of the Pacific [20], the Bantu expansion into southern Africa [7,21] and the colonization of the American continent [22], all of which are rather well dated radiometrically.

A separate issue to the clock calibration, however, is the method used to estimate the age of clades in the tree. Alongside maximum-likelihood and Bayesian approaches, one very simple method that remains widely used is based on the ρ (rho) statistic [23], which measures the average number of mutations from the supposed root of the clade (the ancestral sequence) to each of the sampled sequences in the clade. When divided by the mutation rate for the whole sequence per unit time, it provides an estimate of the age of a given clade in those time units. The mathematics of the approach, and the means by which confidence intervals can be estimated, were explained by Saillard et al. [24] and subsequently implemented in the Network software package (http://www.fluxus-engineering.com/sharenet.htm). However, the ρ approach has received more than its fair share of criticism over the years.

Some of the perceived weaknesses of ρ can, in fact, be seen as strengths. The fact that ρ is a statistic that does not use the tree topology in its calculation, let alone any explicit demographic model, has sometimes been argued to be a drawback, but this feature rather provides a robust and, as we shall see, unbiased estimate against which more assumption-heavy estimates can be compared. In general, with few recent exceptions, whole-mtDNA genome articles published these days rarely use ρ alone but use it alongside maximum likelihood and/or Bayesian inference estimates [21], where it consistently gives similar results. Note though that, as we will discuss below, the topology of the tree has important consequences for the uncertainty of the estimates of age derived from ρ or, for that matter, any other estimator.

However, nearly a decade ago, Cox [25] claimed that the ρ statistic (even when scaled by an appropriate estimate of the mutation rate) produces biased estimates of the time to the most recent common ancestor (TMRCA) of a set of sequences, and that associated confidence intervals do not estimate the uncertainty appropriately. A simple mathematical proof that ρ is unbiased in this context–as, for example, presented by Saillard et al. [24] and Thomson et al. [26], and repeated below–deals with the first claim: any simulation that displays a contradictory result must therefore be flawed. The second claim rests on an expression for the estimated standard error of ρ (equation (3) in [23]) that has not appeared elsewhere.

The ρ statistic has continued to be usefully employed by numerous diverse researchers working across the areas of mtDNA phylogeography [27–30] and disease studies [31], and the use of Y-chromosome [32–37] and X-chromosome [38] variation to study human evolution. Despite this, Cox’s arguments continue to be cited, even in widely-used textbooks covering human evolutionary genetics [39,40], as well as research papers [41] and high-profile reviews [42].

Our present purpose is therefore to: (a) prove again that ρ is unbiased; (b) to try to reproduce some of the simulations performed by Cox [25]; and (c) to illustrate the value of ρ by drawing age estimates from the literature where both ρ and other methods were employed for the same clades and the same datasets. We will (d) also explore the question raised regarding the coverage of confidence intervals derived from ρ.

## Methods

We carried out a simulation aiming for the same conditions as simulations described before that yielded an apparent bias for ρ [23], up to an ambiguity about population and sample sizes being for haploids or diploids (10,000 realizations of a coalescent process, with haploid population size *N* = 1000, haploid sample size *n* = 100, mutation rate across the whole sequence *μ* = 0.00234 per generation [coming from Cox’s assumption of a transition rate of 1.8×10^−7^ per base-pair per year, a generation time of 26 years and a sequence length of 500], giving *ϑ* = 2*Nμ* = 4.68), coded in R [43] (scripts available at http://www.stats.gla.ac.uk/~vincent/rho). Trees were generated using the constant-size coalescent process and mutations assigned to its edges under the infinite sites model.

For comparison of real data, we collected data from several published manuscripts that displayed a comparison between ρ and maximum likelihood [9,10,11,44–47] using the same mtDNA dataset. In order for all the ages to be comparable we used age estimates based only on the time-dependent mtDNA molecular clock we developed [14]. This earlier work also addressed many of the problems also raised by Cox concerning the accuracy and precision of the mtDNA mutation rate, including the issue of selection, and we have also discussed previously how uncertainty in the estimates of the mutation rate can affect the outcomes [10], but the comparison is fully independent of the molecular clock used, as the objective is to compare the methods of estimating branch lengths and not the actual age estimate. ML age estimates in the different studies were performed with PAML [48] using the HKY85 model with gamma-distributed rates. We note that other criticisms of ρ made by Cox, such as mtDNA mutation rate heterogeneity, are addressed by the comparisons with ML, which explicitly takes the tree structure into account; and the issue of mtDNA homoplasy has been addressed in the last decade by focusing on highly informative molecular sequences, such as whole mtDNA genomes, which provide better resolved molecular phylogenies.

## Results and discussion

### Mathematical demonstration

Suppose that the genealogy of a sample of *n* sequences consists of a tree of *m* links, the *j*th of which has length *T*_*j*_ generations and bears *R*_*j*_ mutations. The sets Ψ_*i*_ (*i* = 1,…,*n*) have as members the indices of the links between the MRCA and the *i*th leaf. Let the total mutation rate of the gene segment be *μ*. Then *R*_*j*_|*T*_*j*_ ~ Po(*μT*_*j*_), independently. The statistic *ρ* is the mean number of mutations across all the paths from the MRCA to the leaves: $\rho =\frac{1}{n}\sum_{i=1}^{n}L_{i}$, where $L_{i}=\sum_{j\in \Psi_{i}}R_{j}$. Hence, *L*_*i*_|{*T*_*j*_} ~ Po(*μT*), where *T* is the time to the MRCA (TMRCA), by the reproductive property of the Poisson distribution, and the fact that each path is of length *T*. Note, however, that in general the *L*_*i*_s are not independent (unless the Ψ_*i*_s are disjoint). So $E[\rho ]=\frac{1}{n}\sum_{i=1}^{n}E[L_{i}]=\mu T$. Hence *ρ*/*μ* is unbiased for *T*. This argument goes through whether the *T*_*j*_s are considered random (*e*.*g*., the result of a coalescent process) or fixed parameters (as in the classical phylogenetic setting).

### Comparing age estimates in simulations and real data

**Fig 1:**
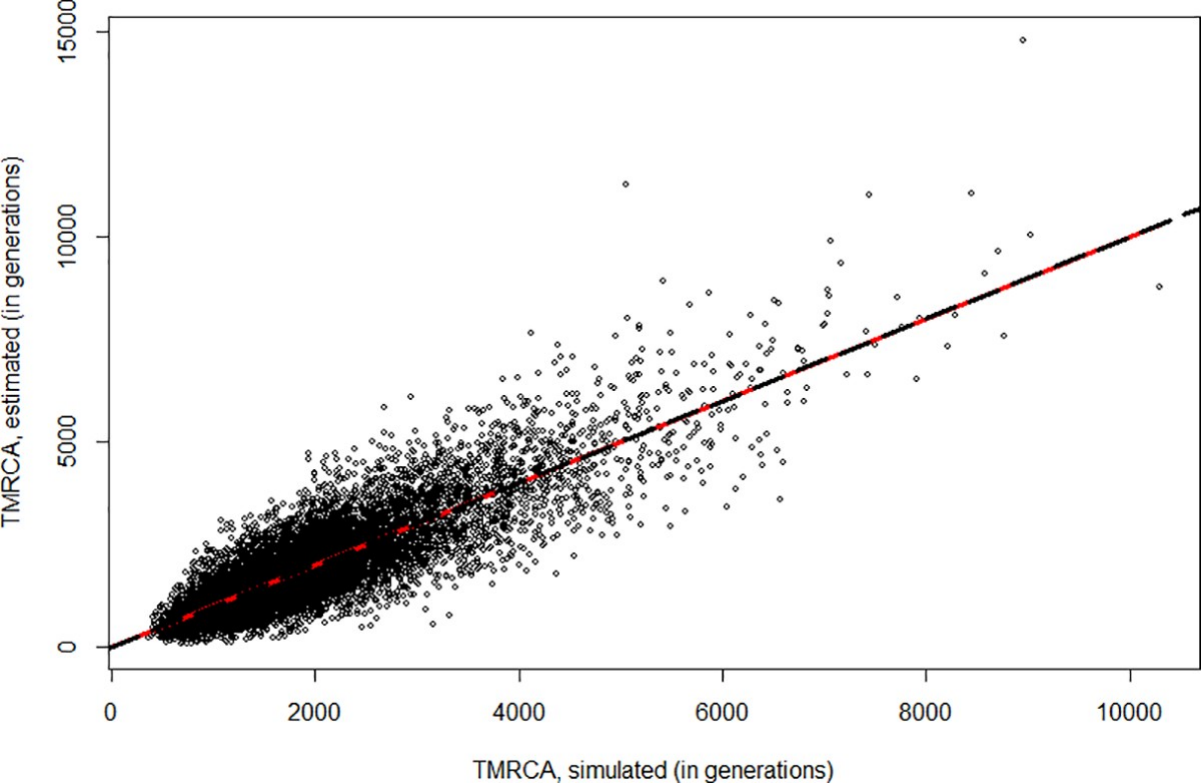
Scatterplot comparing the estimated time to the most recent common ancestor (TMRCA) using *ρ* and the true simulated time, across 10,000 simulations using a constant-size coalescent process. The line of equality (red) and the least-squares regression (black dashes) are superimposed, meaning that estimated TMRCA with *ρ* shows no bias.

In light of the mathematical demonstration, the bias reported by Cox [25] (their Fig 2) is puzzling and we are unable to reproduce it. A simulation aiming for the same conditions as that figure yielded Fig 1, where the TMRCA estimated using *ρ*/*μ* is plotted against the true TMCRA of the simulated trees. The line of equality (red solid line) and the least-squares regression line through the origin (black dashed line) are virtually indistinguishable, and the slope of the regression line is not significantly different from 1. The observed bias in this finite sample of 10,000 runs is just 1.7 generations.

**Fig 2:**
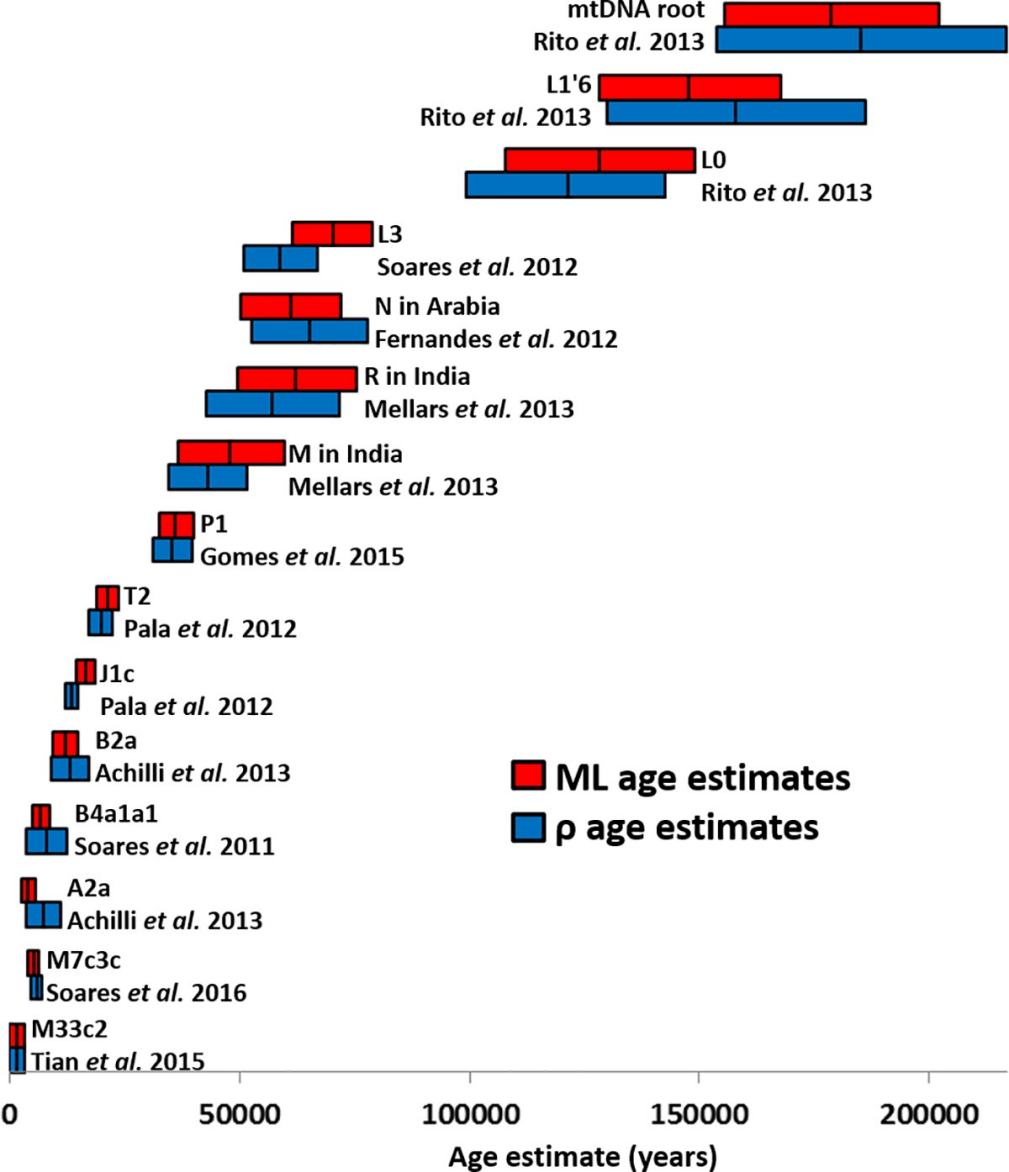
Comparison of age estimates from the literature based on ρ and maximum likelihood (ML) using the molecular clock developed by Soares et al. [14]. ML age estimated were generated using the HKY85 evolution model and gamma distributed-rates using the PAML software [48]. Size of the bars correspond to the 95% confidence interval of each age estimated based in the standard error as obtained by PAML in ML [48] or using the Saillard et al. calculation in ρ estimates [24].

Although there is therefore no evidence that ρ is a biased estimator (and indeed a proof that it is not), we compared age estimates from the literature. We used ages that were estimated with both ρ and ML using the same dataset (Fig 2). It is clear that the mtDNA coalescence age estimates are very similar between the two estimators. The same observation has been made for Y-chromosome variation by Batini et al. [37], when comparing ρ and Bayesian inference. There is no observed trend where the age estimates based on ρ are systematically higher or lower (the latter as suggested by Cox [25]) than ML estimates. A correlation between age estimates using both methods displayed a relationship of nearly 1 (0.9925, R^2^ = 0.9951). Haplogroup L3 is the only one whose age estimates were substantially different between ρ and ML. As previously discussed [8,11], this is due to the high frequency of L3e, associated with the Bantu expansion. However, this in itself shows the random nature of the difference and not a directional bias: if a branch with higher average length like L3a or L3h were the most frequent it could easily lead to an over-estimate in relation to ML.

### Coverage of confidence intervals

The coverage of confidence intervals derived from *ρ* is a pertinent issue, but Cox’s discussion of this is compromised at the outset by his expression (3) for the estimated squared standard error of ρ [25], which, to our knowledge, has never before been used to assess the error in *ρ*. It corresponds neither to the expression proposed by Saillard et al. [24], and used extensively thereafter and herein, which incorporates the dependence of the *L*_*i*_s, nor to the lower bound given by *ρ*/*n*, which assumes a perfectly star-like genealogy for the sequences and as a result can seriously under-estimate the error. Cox’s expression is a halfway house between these two: it corresponds to a tree which is star-like in the distinct *haplotypes*. Most crucially, it does not describe the increased uncertainty arising from the mutations on internal edges of the tree. Or, to put it another way, it assumes the number of mutations from the root to each distinct haplotype are independent random variables, which since the haplotypes are related by a tree, they in general are not.

**Fig 3:**
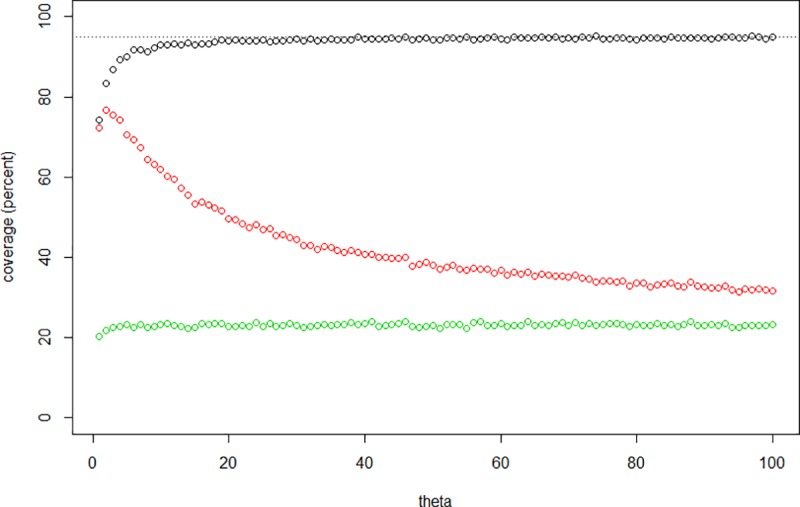
Coverage of Wald confidence intervals for the estimated time to the most recent common ancestor (using *ρ*) as a function of *ϑ*, based on Saillard et al. [24] (black circles), Cox [25] (red circles) and lower-bound (green circles) estimates of standard error, obtained from 10,000 simulations using a constant-size coalescent process. The nominal coverage of 95% is indicated with a dotted line.

We have explored the coverage of the commonly used Wald-style confidence intervals provided by the end points (*ρ* ± 1.96 e.s.e.[*ρ*])/*μ*, where the estimated standard error (e.s.e.) is that given by Saillard et al. [24]. Given that this expression reflects the shape of the underlying genealogy, which is itself influenced by the demography, we should expect different coverage properties under different demographic scenarios. Fig 3 illustrates the estimated coverage as a function of *ϑ* in the simplest, constant-size, model described above (with 10,000 realizations at each *ϑ* value). Coverage is indeed anti-conservative for small values of *ϑ* (when the sampling distribution of *ρ* is very skewed), but is acceptable if *ϑ* ≳ 10. Note, however, the opposite behaviour of the coverage of the Wald confidence intervals derived from Cox’s expression for estimated standard error: it decreases with increasing *ϑ*, and is always smaller than the Saillard coverage. Not surprisingly, the coverage provided by the lower bound on the standard error, mentioned above, is everywhere extremely poor.

There is no denying that the precision with which *ρ*/*μ* estimates the TMRCA depends on the (unknown) demography, since the demography influences the correlation of the *L*_*i*_s (via the tree) and hence the standard error of *ρ*. A demography that leads to long internal edges in the tree (e.g. constant population size) will lead to much more correlation between certain *L*_*i*_s and hence to data sets that have much less information about the TMRCA, whereas star-like trees (e.g., coming from population expansion) lead to much less correlation and to more informative data sets. No method of estimation could (or should!) get around that. Hence it is doubly important to have a method of estimating the standard error of *ρ* that accounts well for the correlation of the *L*_*i*_s, as the Saillard standard error estimator does, and Cox’s does not (since they are assumed either to be perfectly correlated if they lead to the same haplotype or independent if they lead to distinct haplotypes, and nothing in between).

In this paper we have, like Cox, explored the variability in ρ. The transformation of ρ into an estimate of the age of a node in the phylogeny requires division by a well-calibrated mutation rate. Obtaining this is not a trivial task, and much effort has been invested in it over the years. Any calibration should, at the very least, supply some quantification of error in the estimated mutation rate. Given that, say in the form of an estimated standard error, the delta method [49] provides a quick route to a crude estimate of the standard error of the age of the node of interest [10].

## Conclusions

In summary, we have shown that ρ is an unbiased estimator through a mathematical proof; but we additionally supported this conclusion by means of simulations and, empirically, by comparing age estimates for clades simultaneously obtained through ρ and ML. We have also shown that the coverage of the confidence intervals is only problematic for lower values of *ϑ*, contrary to previous suggestions. Overall, this shows that ρ should not be dismissed, as suggested; it can play an important role in genetic dating. This is a crucial first step in many lines of research based on phylogenetic analysis, but it is only the first step–discussion of how the estimated dates of nodes in a tree can be interpreted, for example in drawing conclusions about gene flow and population history, is a much larger topic [19].
